# Assessing spatial equity in integrated health care facilities for older adults: a case study in the metropolis of Guangzhou, China

**DOI:** 10.3389/fpubh.2026.1890540

**Published:** 2026-07-13

**Authors:** Yingsheng Liu, Changdong Ye, Yifei Wu, Kailun Fang, Jiyang Mi

**Affiliations:** 1 College of Forestry and Landscape Architecture, South China Agricultural University, Guangzhou, China; 2 Simon Fraser University, Burnaby, BC, Canada; 3 Key Laboratory of Natural Resources Monitoring in Tropical and Subtropical Area of South China, Ministry of Natural Resources, Guangzhou, China; 4 School of Architectural Engineering, Shenzhen Polytechnic University, Shenzhen, China; 5 Guangzhou Urban Planning and Design Co., Ltd., Guangzhou, China

**Keywords:** Guangzhou, integrated health care facilities, older adults, spatial equity, two-step floating catchment area method

## Abstract

**Background:**

Ensuring equitable access to health care facilities for older adults is an increasingly important concern for academic researchers and policymakers in building age-friendly cities and communities. Previous studies have seldom explored the spatial equity in the distribution of geographic access to multi-tiered and multi-type health care facilities in China, especially community-based care for older adults.

**Methods:**

To compensate for this gap, this article adopted the two-step floating catchment area (2SFCA) method to systematically and dynamically evaluate equity in spatial accessibility by accounting for the category and hierarchy of health care facilities in Guangzhou by investigating the accessibility among 176 towns/districts and 973 health care facilities that were classified by municipal and community levels as well as medical facilities and nursing facilities in six different years.

**Results:**

The results suggested that the supply-demand relationships were regionally unbalanced in Guangzhou. The supply of all types of health care facilities in the suburban area was optimistic, whereas that in the central city was inadequate considering its high density of older adults. Accessibility on the outskirts was complex, and areas with insufficient supply. Moreover, community-level and nursing facilities had lower accessibility than municipal-level and medical facilities.

**Conclusion:**

Along with aging in the coming three decades, the supply-demand relationships of all health care facilities will lag in terms of supply. These findings may provide helpful insights for urban planners, and public officials with respect to the equal provision of health care resources and site allocations for older adults.

## 1 Introduction

The dramatic increase in the aging population is engendering a shift in population distribution toward an aging society (1). Definitions of older adults age vary, with WHO and UN statistics reporting both 60+ and 65+ populations; however, 65 and over is more commonly used as the standard threshold in UN studies. According to data compiled by Our World in Data, around 830 million people were aged 65 and above worldwide in 2024 (2). The United Nations projects that this number will more than double to 1.6 billion by 2050. Older adults will then make up over 16% of the global population (3). This rapid demographic shift calls for a concerted effort to create ‘age-friendly cities and communities', which was initiated by the WHO, where older adults have equitable access to health care services (4). As the world's most populous country, China has experienced a dramatic increase in the older adults in recent years. According to China's National Bureau of Statistics, the proportion of people aged 65 and above in China reached 14.2% of the total population in 2020 (5). In response to this aging trend and the upcoming extension of the retirement age. The Chinese government has implemented various strategies to promote the equalization of health care services and improve their quality of life (6). However, the older adultsfrom different areas or different socioeconomic groups also encounter considerable disparities in health care services, which has important spatial/social equity implications for policy-makers (7).

Likewise, in fields such as public health, economics, and geography, researchers have demonstrated longstanding contributions to the optimization of health care services (6, 8). Although many studies have suggested that efficiency and equality are the most important objectives for allocating health care resources, most of the literature focuses on the nonspatial perspective, including health policy reform, income status, service capacity and quality (9). Few studies have investigated the provision of health care services from the spatial perspective, especially in the context of Chinese health care service reform (10). Specifically, the Chinese central government implemented the Hierarchical Diagnosis and Treatment reform in 2015 with a series of strategies, aiming to improve the efficiency and equity of multilevel health care (11). One of the government's strategies is to integrate community-based old-age nursing care with medical care by introducing an integrated health care system (12). Accordingly, the Chinese government has designated 90 national pilot cities to develop this new system (6)However, very little has been written to explore the spatial equity in the distribution of geographic access to integrated health care facilities (including multi-tiered and multitype facilities) in China, especially community-based nursing care for the older adults, which hinders the more reasonable allocation of health resources in spatial and land use planning (13).

This study attempts to probe into the provision of Chinese integrated health care facilities through an analysis of the accessibility disparities in terms of service supply and demand. The objective is fourfold, namely, to (1) demonstrate the spatial characteristics of an aging population based on various geographical areas in the last two decades; (2) reveal the accessibility of integrated health care facilities at the town/street level; (3) analyze the spatial stratification of health care facilities by classifying six types from the supply and demand perspective; and (4) provide suggestions for further health care supply based on a population prediction model for the next consecutive decades.

The following part of this paper is organized into five sections. It begins with a critical review of existing academic inquiries into the issue concerning China's growing aging population and ongoing health care reform. This is followed by a clarification of methodological issues, including the definition of key concepts, study area, chosen reasons, data sources and analysis method, as well as research design. Attention is then turned to an empirical investigation of Guangzhou as an important case to examine the provision of integrated health care at the town/street level in sections 4 and 5. The last part sums up the main findings and discusses implications for future studies and policy-making.

## 2 Theoretical context

### 2.1 The development of integrated health care in China

In the late 1970s, China embarked on economic reforms, leading to a transition from a centrally planned economy to a market-oriented system. This period saw changes in health care delivery, including the introduction of private health care providers and a focus on improving medical technology and infrastructure. In recent years, China has faced the challenges of an aging population, with a growing number of older adults requiring health care and long-term care services. The government recognized the need for a more comprehensive and community-based approach to older adults care by incorporating social groups. As a result, the Chinese government has been focusing on developing policies and initiatives that aim to integrate old-age care and medical care more effectively, shifting the focus from institutional care to home and community-based alternatives (6, 14). The goal is to provide seamless and coordinated services that improve the overall wellbeing and quality of life for older adults.

One of the important reforms is to promote community-based care services and facilities, such as home-based care^1^ (15), daycare centers^2^ (16), and community health centers^3^ (17). With the provision of health care services at different levels and types, this approach allows older adults to receive appropriate care and support while remaining in their familiar environments and close to their families (12). However, due to the lack of qualified caregivers, insufficient organized funding, and resource provision, community-based health care facilities are inadequate, and the older adults do not have equal access to them (14). In this context, it is important to explore evidence-based policies to guide health care facility administrators and policy-makers in making informed decisions to benefit the broader population with effective and equitable resource delivery in the development of integrated health care.

### 2.2 Research on the aging Chinese population

Recent decades have witnessed a drastic growth in the aging population in China, which has aroused heightened public attention and scholarly interest. Given the social importance of the subject matter, many scholars have made extensive efforts to understand the uneven access to health care services and provide suggestions for equal allocation of health care coverage and facilities. Most existing research on public health service facilities has primarily placed its emphasis on the nonspatial perspective, including financial access (e.g., payment capacity of various social groups, provision of medical insurance, costs of health care utilization, shortage of sustainable funding), service capacity (e.g., adequacy of health service provision such as the number of physicians, equipment, and hospital beds) and provision quality (e.g., appropriateness and suitability of health services, weak quality regulations) (9). For example, a theoretical study of China's long-term care system published in The Lancet revealed that workforce shortages and weak quality assurance are challenges in long-term care services, especially the slow development of home and community-based services (6). Other nonspatial factors, such as inefficient delivery, low quality of care, and control of health expenditures, have also been (11).

Apart from the nonspatial perspective, a small number of studies conducted by medical geographers and urban geographers have stressed the spatial access of health care facilities, along with the important progress of geographic information systems (GIS) and the availability of spatially disaggregated data on both health care supply and demand (18). In these studies, professional analysis of service provision from a geographical perspective is the most direct and effective way to resolve problems associated with low accessibility and uneven allocation (19). With a consensus on the major influence of the built environment on public behaviors and health care outcomes, these scholars have employed the GIS methods to measure the spatial accessibility of health care facilities to optimize the equal provision of health care services (18, 20). Recent research has also incorporated temporal dynamics into accessibility assessments. For example, Niu et al. (21) examined the dynamic accessibility and spatial equity of emergency medical services for older adults in Zhengzhou using a 2SFCA-based approach.

Despite their different analytical angles and perspectives, previous studies in China have mostly treated healthcare demand as uniform. Although they consider hierarchical facilities on the supply side, they rarely account for the different needs of older adults or for how multi-level services interact with varied demand (7, 22). In reality, in response to China's heavy burden of medical resources, the Chinese government has put forward strategies to provide different qualities and types of health care services, namely, medical hospitals, general care facilities, community-based medical institutions and nursing care, to guide older adults' health-seeking behaviors in different situations (12). Specifically, people tend to go to medical facilities (MFs), such as general hospitals (GHs) and community hospitals (CHs), for diseases or nursing care facilities (NFs), such as municipal rest homes (MHs) and home-based pension institutions (HPs), for normal health care. Even for the same type of care service facilities, there are variations in their level to meet residents' different needs. As community-level facilities, CHs and HPs can meet people's basic needs for daily health treatment when they are not seriously ill, whereas when people have severe injuries or critical diseases, municipal-level facilities, such as GHs and MHs, are needed.

However, the literature is surprisingly lacking in the systematic evaluation of geographical equity in the distribution of access to integrated health care facilities in China (23). Even though some researchers have differentiated the levels of health care facilities, they often assume older adults to be a homogenous group with little attention to the diversity of their circumstances and needs. Even for limited research differentiating the levels of health care facilities, studies have focused only on a single level of health care, such as primary care (24, 25). A national study on spatial accessibility to primary health care centers demonstrated greater inequalities in the north and northeastern provinces and identified communities with lower proximity and accessibility to primary health care (24). This disparity in access to primary health care is also apparent in empirical research in Sichuan Province, China (25). Some studies have focused only on medical facilities while overlooking another important category of health care facilities, namely, nursing care facilities. As emphasized by Feng et al. in their research on China's long-term health care services, the extent to which existing health care facilities meet older adults' needs is unclear due to insufficient data and evaluation of their accessibility. Without seriously investigating the actual practices and effects of Chinese hierarchical health care systems, it would be difficult to unveil the picture showing the efficiency and distributional aspects of the recently advocated policy and provide valuable suggestions for policy-makers.

## 3 Data and methodology

### 3.1 Study area

The study area was Guangzhou, the third largest city after Beijing and Shanghai, with a total population of 18.67 million in 2020. As the city that developed the earliest after the reform and opening up policy and with a location in the southeast coastal area of China, Guangzhou has experienced consistent migration since the 1990s, and many of these people are aging. Population aging has become increasingly serious in recent years. The proportion of older adults in 2000 was 6.1% and rose to 7.82% by 2020. According to the seventh census data, the number of people aged above 60 and 65 was 2.13 million and 1.46 million, accounting for 11.41% and 7.82% of total people, respectively (5, 26). This demographic shift has prompted the need for comprehensive and accessible health care services that cater to the specific needs of older adults. In this study, older adults were defined as individuals aged 65 years and above, consistent with the age threshold commonly adopted by the United Nations in population aging statistics and old-age dependency indicators.

To address the challenges posed by the aging population, a series of public policies has been issued successively by the municipal government in Guangzhou, which aims to enhance the professionalism of health care workers, optimize the allocation of health care resources, and meet older people's health needs (13, 27). One of the important initiatives is to offer a range of medical and nursing care facilities, including nursing homes, welfare centers for older adults, older adults care centers, and community health centers. Despite certain achievements and relatively abundant medical resources, it is not clear how and to what extent the practice of integrated health care, especially community-based facilities, meets the demand of Guangzhou's sizeable aging population. Therefore, the selection of Guangzhou as the study area is of great importance.

**Figure 1:**
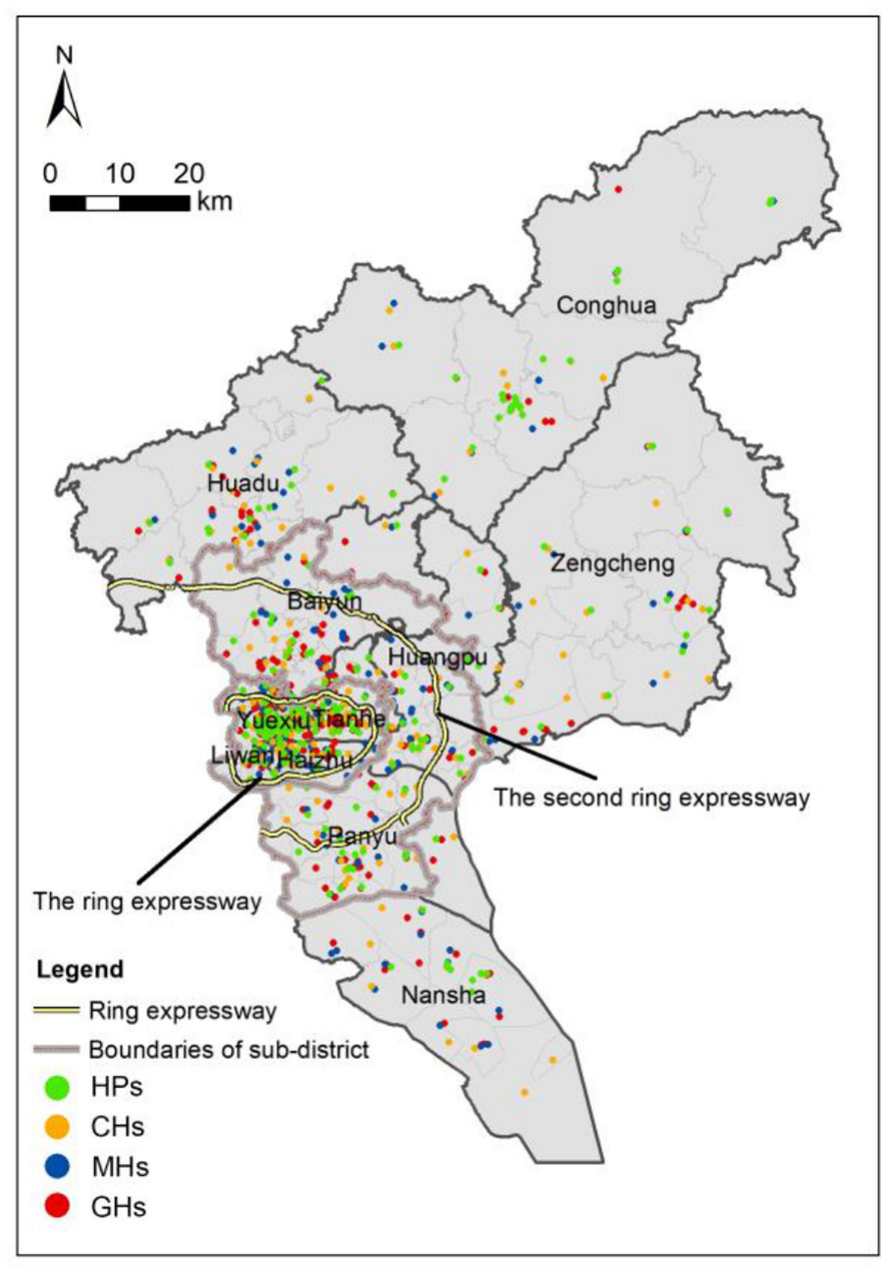
The study area.

Our research covered the entire city area, which includes 11 administrative districts and 176 towns/streets (Figure 1). We divided the study area into three subdistricts based on their age, population density, administrative boundaries, and two ring expressways, as shown in Figure 1. The core subdistrict is the central area, which includes 81 towns in the entire Haizhu, Liwan, Yuexiu, and partial Baiyun and Tianhe administrative districts. Most of this area was built before 2000, mainly within the ring expressway, and has the highest population density of 21,360 people/km^2^. The second subdistrict is the suburban area of the city, which has both urban and rural characteristics. The area includes 49 towns in partial Baiyun, Huangpu, Tianhe, and Panyu administrative districts. Most of this area was built after 2000, mainly within the second ring expressway, and has a moderate population density of 5,616 people/km^2^. The third subdistrict is the outskirt area in the periphery, which mainly displays rural characteristics. The area includes 46 towns in the entire Conghua, Huadu, Nansha, and Zengcheng districts and partial Baiyun, Huangpu, and Panyu administrative districts. The population density is the lowest at 914 people/km^2^.

### 3.2 Data

The data used in our research include demographic and health care facility data. Demographic data include data from the fifth, sixth, and seventh population censuses in 2000, 2010, and 2020, respectively. Each census has town-level population numbers of people aged 0–14, 15–64, and above 65; in particular, the seventh population census has year-by-year population numbers, thus helping to predict population aging for the future. Older adults are defined as people over 65 years of age.

**Figure 2:**
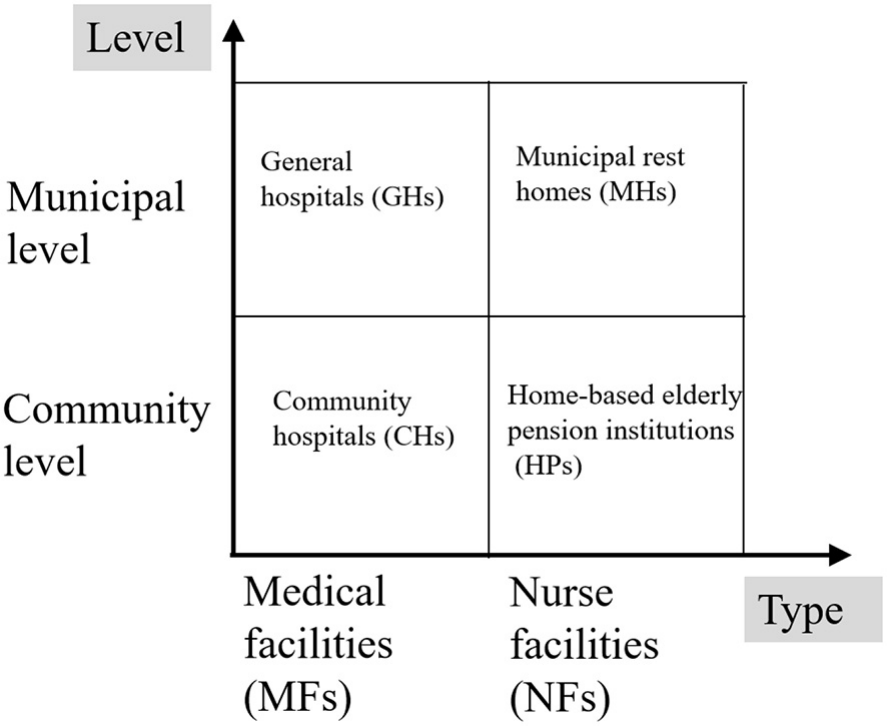
Four types of health care facilities categorized by level and type.

Health care facility data come from the Guangzhou Municipal Health Commission. The health care facilities for older adults include two groups of medical facilities (MFs) and nursing care facilities (NFs), which consist of community-level facilities for basic services and municipal-level facilities for critical or specific services. Medical facilities are community hospitals (CHs) for common diseases and general hospitals (GHs) for critical diseases, while community-level and overall-level nursing care facilities are home-based pension institutions (HPs) for daily care and municipal rest homes (MHs) for professional care (Figure 2). Since CHs and HPs were first built in 2009 and 2016, there are no data on CHs in 2000 or HPs in 2000 or 2010 (Table 1). These are both public and private institutions. The location and number of hospital beds, which represent the service capacity of each health care facility, were included in our research data. Most of older adults' health care facilities were distributed in the central area and were less concentrated from the central area to suburban and outskirt areas, according to population density.

**Table 1:** Data on older adults' health care facilities from 2000 to 2020.

Sub district	Indices	2000			2010		2020			
		Nursing care facilities	Medical facilities	Nursing care facilities	Medical facilities		Nursing care facilities		Medical facilities	
		MHs	GHs	MHs	GHs	CHs	MHs	HPs	GHs	CHs
Central area	N	5	81	41	101	43	103	112	141	78
	Beds	1,188	58,260	8,983	65,726	2,080	20,532	2,240	74,954	3,780
Suburban area	N	15	51	24	64	19	64	60	99	50
	Beds	5,492	24,335	12,599	30,687	1,020	24,366	1,200	43,749	2,680
Outskirt area	N	17	39	23	46	24	60	83	63	60
	Beds	2,659	15,079	4,232	17,449	1,340	17,781	1,660	22,575	2,960
Entire city	N	37	171	88	211	86	227	255	303	188
	Beds	9,339	97,674	25,814	11,3862	4,440	62,679	5,100	141,278	9,420

### 3.3 Methods

First, we used the older population, population aging rate, and population aging speed to display the strength, extent, and speed of population aging, respectively. The population aging speed contains two time intervals, 2000–2010 and 2010–2020, and the other two contain three years, 2000, 2010, and 2020. By comparing these three indices of population aging, we depicted a longitudinal temporal change and its spatial pattern of population aging in the last 20 years.

**Figure 3:**
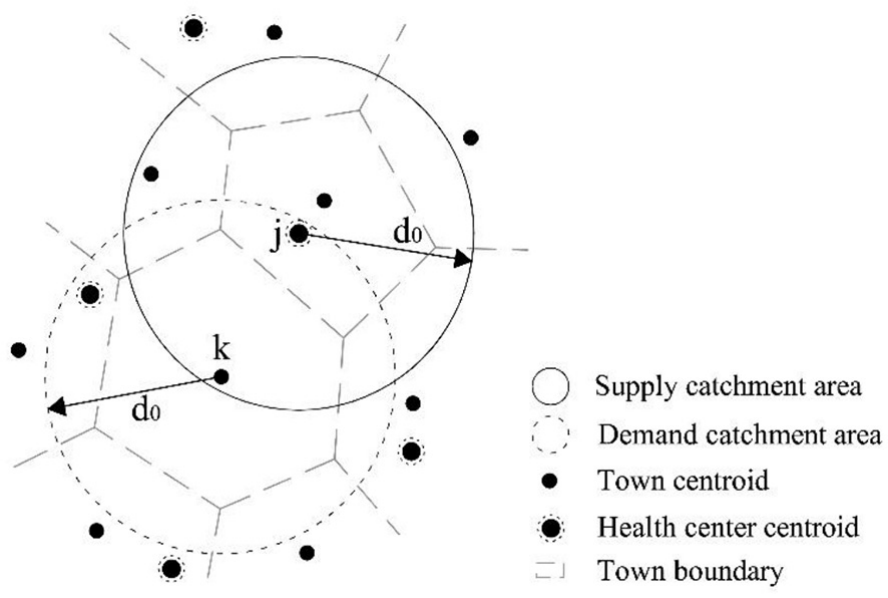
The concept of the two-step floating catchment area method.

Next, we used the 2SFCA method to analyze the spatial accessibility of health care facilities for older individuals. The method was first developed by Luo (28) to assess the spatial accessibility of physicians and was extended to calculate the spatial accessibility of different facilities in many studies (23, 29). The concept of the 2SFCA method is shown in Figure 3; it takes both the demand side of older adults and the supply side of health care facilities into consideration by using a distance decay function.

The calculation process of the 2SFCA method is as follows (30, 31): the first step is to calculate the supply ratio (R) of health care facilities as Formula (1) by the Gaussian distance decay function (2). The second step is to calculate the spatial accessibility of older adults in different towns using Formula (3).


$$\begin{array}{l} R_{j}=\frac{S_{j}}{\underset{k\in /d_{kj}\leq d_{0}\;}{\sum }G\left(d_{kj},d_{0}\right)P_{k}} \end{array}$$ (1)

$$G(d_{kj},d_{0})=\{\begin{array}{ll} \frac{e^{-\frac{1}{2}\times {\left(\frac{d_{kj}}{d_{0}}\right)}^{2}-e^{-\frac{1}{12}}}}{1-e^{-\frac{1}{2}}}\;, & if\;\;\;d_{kj}\leq d_{0}\\ 0, & if\;\;\;\;\;d_{kj}\geq d_{0} \end{array}$$ (2)

$$\begin{array}{l} A_{k}=\underset{j\in \left\{d_{kj}\leq d_{0}\right\}}{\sum }{G\left(d_{kj},d_{0}\right)R}_{j} \end{array}$$ (3)

where *R*_*j*_ is the supply ratio of health care facility *j*. *S*_*j*_ is the service capacity of health care facility *j*, which we use hospital bed number to represent. *D*_*kj*_ is the distance of health care facility *j* to the center of town *k*. *d*_0_ is the distance threshold of different types of health care facilities, which is flexibly defined in the 2SFCA framework and has no universal standard, and should be determined based on local travel behavior and empirical evidence (32). Following previous studies that adopt time-based catchments (e.g., 10–60 min or 30 min thresholds) for healthcare accessibility (33, 34), defined as HPs and CHs at a travel time of 10 mins with comfortable travel; people generally walk to HPs, so *d*_0_ of HPs is approximately 600 m; people mostly drive to CHs, so the *d*_0_ of community health care centers is approximately 3.33, 6.66 and 10 km in central, suburban and outskirt subdistricts, respectively, according to the respective average driving speed of 20, 40 and 60 km/h; and the *d*_0_ of MHs and GHs uses a travel time of 30 mins. *Thus, d*_0_ is approximately 10, 20 and 30 km in the three subdistricts, respectively. *P*_*k*_is the older population in town *k*. *G(d*_*kj*_*, d*_0_*)* is the distance decay coefficient between health care facility *j* and town *k* by Gaussian function. *A*_*k*_ is the spatial accessibility of older adults to health care facilities in town *k*.

Next, we classified the population aging of different towns/streets of Guangzhou into six types in 2020 according to the supply-demand relationship to further understand the population aging status and the demand for health care in different areas. The supply-demand relationship was examined based on the accessibility of health care facilities for older adults and the density and degree of aging in different towns and streets. Specifically, the density of aging was defined as the number of older adults divided by the administrative area of the township, and the degree of aging was defined as the number of older adults divided by the total population of the township. It was assumed that the accessibility data calculated in the previous step represent the supply capacity of health care facilities for older individuals, and two aging indices represent the demand, according to which we judged the matching relationship between the supply and demand level of health care facilities in each town and street in 2020. Based on this relationship, each town/street was categorized into six types, as shown in Table 2: high-level supply-demand balance, oversupply, undersupply (which can be further classified into three types, density-oriented, degree-oriented, and bifactor-oriented), and low-level supply-demand balance (35).

**Table 2:** Types of supply–demand matching relationships for health care facilities for older adults in 2020.

Type code	Type	Supply level (2020)	Demand level (2020)
11	high–level supply–demand balance	Accessibility ≥ mean	At least one index of density and degree ≥ mean
21	oversupply	Accessibility ≥ mean	Both density and degree indices < mean
31	density–oriented undersupply	Accessibility < mean	Density index ≥ mean
32	degree–oriented undersupply		Degree index ≥ mean
33	bifactor–oriented undersupply		Both density and degree indices ≥ mean
41	low–level supply–demand balance	Accessibility < mean	Both density and degree indices < mean

Finally, we used the age-shifting algorithm to predict population aging in 2030, 2040, and 2050. The projection is based on the full age structure of the 2020 baseline population. We considered both the death rate and migration rate in the prediction model, calculated as Formula (4):


$$\begin{array}{l} P_{t}=\overset{\left(n-65\right)}{\underset{i=0}{\sum }}P_{\left(65-t+i\right)}\left(1-D_{\left(65-t+i\right)}\right)\times {\left(1+M_{\left(65+\right)}\right)}^{t} \end{array}$$ (4)

$$\begin{array}{l} M_{65+}=TP_{65+}-NG_{65+} \end{array}$$ (5)

$$\begin{array}{l} TP_{t}=TP_{0}\times {\left(1+G\right)}^{n} \end{array}$$ (6)

where *P*_*t*_ is the number of people aged over 65 in the next t years, P (65-t+i) is the number of people aged (65-t+i), D(65-t+i) is the death rate of people aged (65-t+i), *M*_(65+)_ is the migration rate of people aged over 65, i is the yearly age group over 65, and n is the oldest age. *TP*_65+_is the total older population growth rate, and *NG*_65+_ is the natural older population growth rate. *TP*_*t*_ is the total population in the next t years, *TP*_0_ is the total population of the original year, and G is the composition population growth rate.

Based on the predicted population data, this study analyzed the changing demand for health care facilities over the next 30 years in 2030/2040/2050. The accessibility of facilities for older adults in 2020 was used to represent the current level of supply, and two aging indices (density and degree of aging) in the corresponding year were used to represent the future growth trend, thereby providing evidence for further planning suggestions for facility supply. Specifically, there were six types, as shown in Table 3: strong demand (the current supply is high, and the future growth trend is high, during which the level of supply of health care facilities for older adults should be further optimized and improved based on the future situation), oversupply (the status quo supply is high, but future growth trends are not significant; the supply of health care facilities for older adults could be appropriately delayed), lagging supply that can be further categorized into three types of density-oriented, degree-oriented, and bifactor-oriented types (the current supply is not high, but future growth trends are high, and the supply should be increased in the future), and low demand (the current supply and future growth trend are not significant, and the supply could be appropriately delayed.

**Table 3:** Types of supply–demand matching relationships for health care facilities for older adults in 2030/2040/2050.

Type code	Type	Supply level (2020)	Demand growth level (2030/2040/2050)
11	Strong demand	Accessibility ≥ mean	At least one index of density and degree growth ≥ mean
21	Oversupply	Accessibility ≥ mean	Both density and degree growth < mean
31	Density–oriented lagging supply	Accessibility < mean	Density growth ≥ mean
32	Undersupply lagging supply		Degree growth ≥ mean
33	Bifactor–oriented lagging supply	Both density and degree growth ≥ mean
41	Low demand	Accessibility < mean	Both density and degree growth < mean

## 4 Results

### 4.1 Dynamic changes in the aging population from 2000 to 2020

**Figure 4:**
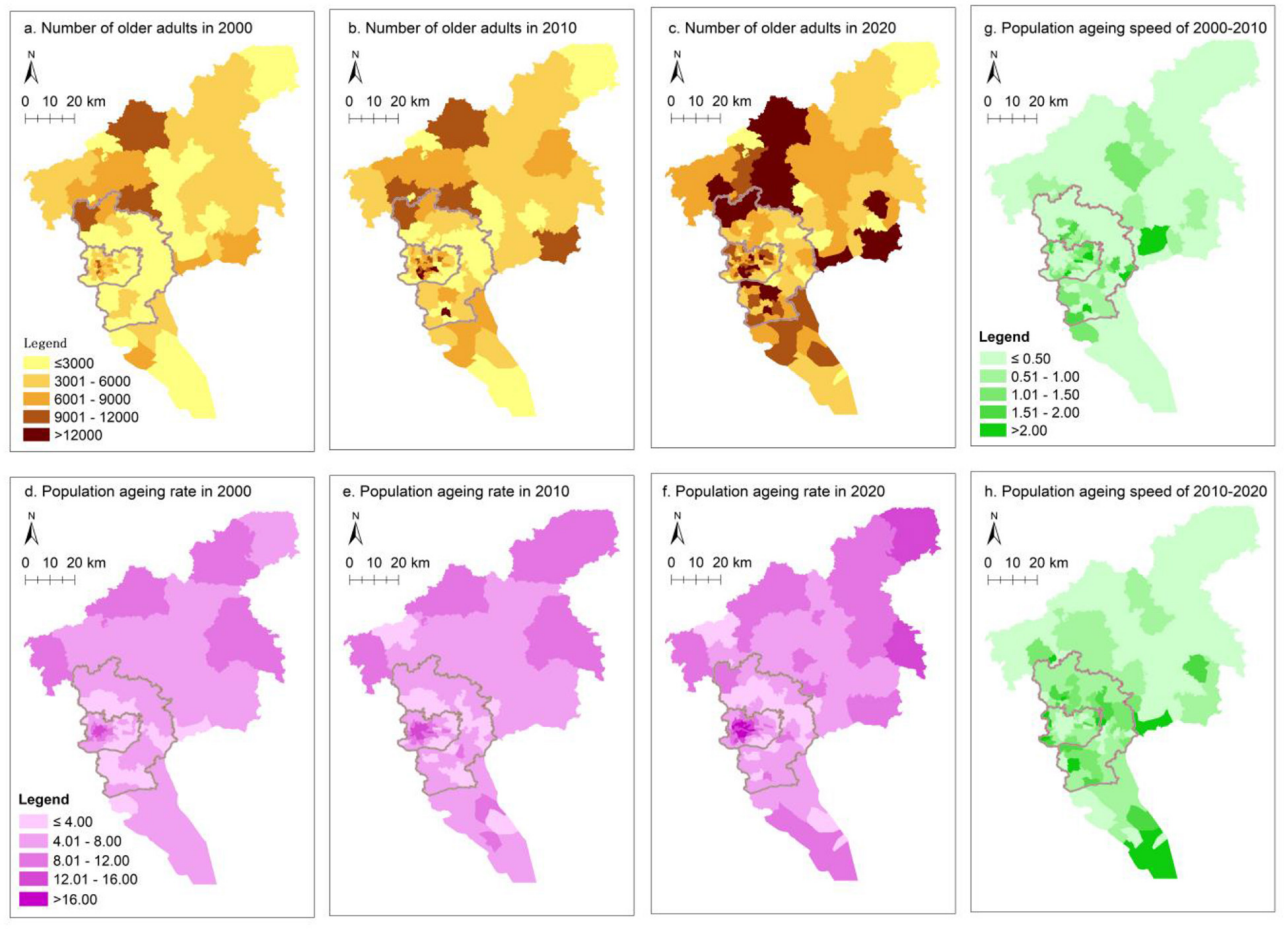
The spatial distribution of three population aging indices in Guangzhou from 2000 to 2020.

Table 4 shows the evolution of the three population aging indices in Guangzhou from 2000 to 2020, and Figure 4 shows the distribution change of the three population aging indices in Guangzhou from 2000 to 2020.

**Table 4:** Indices of population aging in Guangzhou from 2000 to 2020.

Sub district	Number of older people			Population aging rate (%)			Population aging speed (%)	
	2000	2010	2020	2000	2010	2020	2000–2010	2010–2020
Central area	330,602	461,145	668,999	7.46	8.52	9.97	39.49	45.07
suburban area	103.128	158,273	300,684	3.91	4.30	4.55	53.47	89.98
Outskirt area	172.429	228,112	355,474	6.00	6.32	6.65	32.29	55.83
Entire city	606.159	847,530	1,325,157	6.10	6.67	7.82	39.82	56.36

#### 4.1.1 Strength of the central-outskirt pattern: size of the older population

The older adults has grown from 606,159 in 2000 to 1,325,157 in 2020. Older adults are mainly distributed in the central area, with a proportion of more than 50%, while an increasing number of older adults will live in the suburban area during the next 20 years. The respective proportions in the central area, suburban area and outskirt area were 54.54%, 17.01%, and 28.45% in 2000, which changed to 50.48%, 22.69%, and 26.83%, respectively, in 2020. Most towns/streets that have a large number of older adults are in central or outskirt areas, and few of them are located in suburban areas (including a few in the south of Panyu).

#### 4.1.2 Degree of the central pattern: proportion of the older population

The population aging rate increased from 6.10% in 2000 to 7.81% in 2020 for the entire city. The central area displayed the greatest degree of population aging, with an average 2.02% higher than that of the entire city, and the average population aging rate almost reached 10% in 2020; 40 towns/streets compared to 42 in the entire city with the highest population aging rate of more than 12% were located in the central area in 2020, and the other 2 towns/streets were distributed in the outskirts. The suburban area had the lowest population aging rate, indicating that the population age structure here was young with a large labor force, and the population aging rate was less than 5% in 2020. The outskirts had a moderate population aging rate of 6.00% in 2000, which gradually increased to 6.32% in 2010 and 6.65% in 2020.

#### 4.1.3 Speed of the suburban pattern: increase in population aging

The population aging speed accelerated by 39.82% during 2000–2010 to 56.36% during 2010–2020. The speed changes in the central area grew smoothly from 39.49% during 2000–2010 to 45.07% during 2010–2020, which was slower than that in the entire city and the slowest during 2010–2020. The suburban area experienced the fastest growth speed during both 2000–2010 and 2010–2020, and many of the towns/streets here experienced double or even triple older adults growth. The outskirt area showed a similar speed change to that of the central area, with a slower growth speed compared to the entire city of 32.39% during 2000–2010 and 55.83% during 2010–2020.

### 4.2 Spatial accessibility of older adults health care facilities from 2000 to 2020

**Figure 5:**
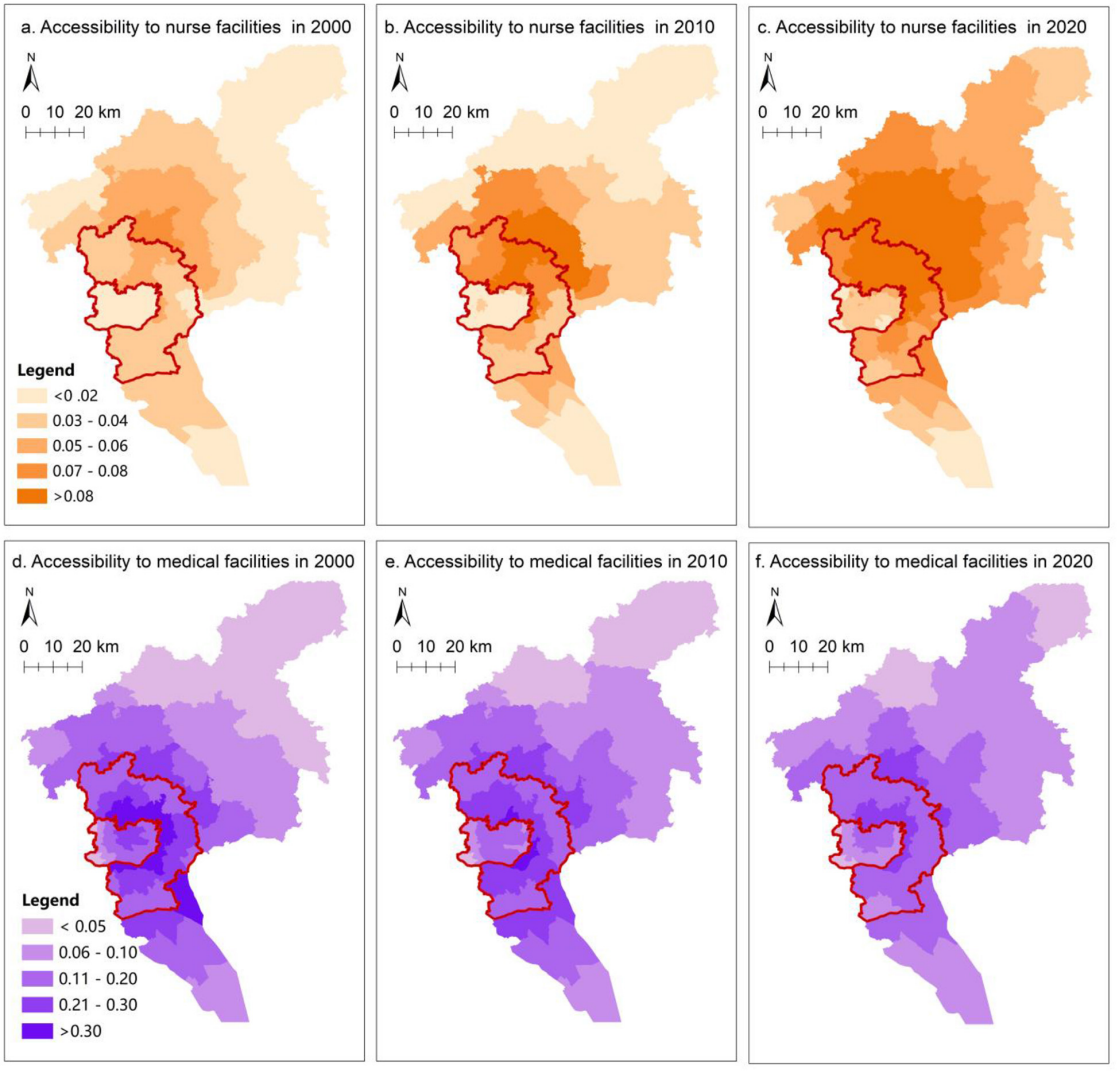
Accessibility of nursing and medical facilities from 2000 to 2020.

**Figure 6:**
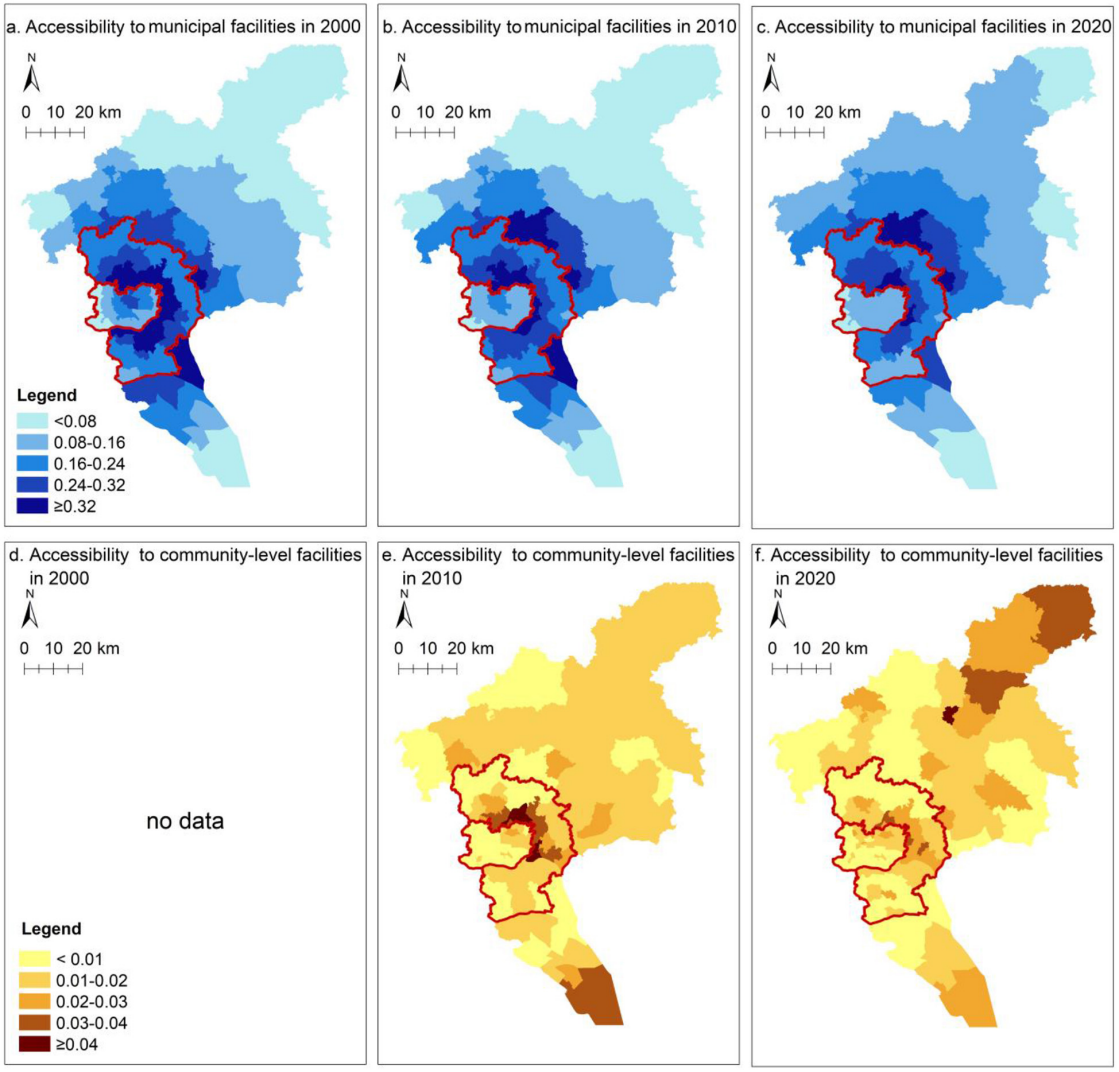
Accessibility of state- and community-level older health care facilities from 2000 to 2020.

Table 5 and Figures 5,6 show the results of older adults health care facility accessibility in Guangzhou from 2000 to 2020. The main characteristics include the following:

**Table 5:** Accessibility of older nursing care and medical facilities in subdistricts from 2000 to 2020.

Year	Facilities	Central area	Suburban area	Outskirt area	Entire city
2000	nursing care facilities (MHs)	0.004	0.033	0.028	0.018
	Medical facilities (GHs)	0.150	0.271	0.130	0.178
	State facilities (MHs+GHs)	0.153	0.304	0.158	0.196
2010	nursing care facilities (MHs)	0.017	0.059	0.039	0.035
	Medical facilities (GHs+CHs)	0.121	0.229	0.135	0.155
	State facilities (MHs+GHs)	0.130	0.267	0.160	0.176
	Community–level facilities (CHs)	0.008	0.021	0.015	0.013
2020	nursing care facilities (MHs+HPs)	0.029	0.080	0.072	0.055
	Medical facilities (GHs+CHs)	0.093	0.171	0.108	0.119
	State facilities (MHs+GHs)	0.113	0.236	0.165	0.161
	Community–level facilities (CHs+HPs)	0.009	0.016	0.015	0.013

First, two kinds of facilities show a reverse direction: nursing care facility accessibility increased quickly from 0.018 in 2000 to 0.035 in 2010 and 0.055 in 2020, while access to medical facilities declined slightly from 0.178 in 2000 to 0.155 in 2010 and 0.119 in 2020. These results indicate that nursing services for older adults largely improved from 2000 to 2020, while medical services did not meet the demand of the growing older population. Second, across three subdistricts, the suburban area had the highest accessibility to both nursing and medical facilities, generally 3 times that of the central area and slightly higher than that of the outskirt area in nursing care facilities, and 2 times that of both the central and outskirt areas in medical facilities. The central area experienced dramatic changes, including both a great increase in nursing care facility accessibility from 0.004 in 2000 to 0.017 in 2010 and 0.029 in 2020 and a large drop in medical facility accessibility from 0.150 in 2000 to 0.121 in 2010 and 0.093 in 2020. The outskirt area showed mild changes in both nurse and medical facility accessibility.

Third, despite the small number of CHs and HPs because they both started in recent years, these facilities helped to reduce spatial disparity by providing more equal services across three subdistricts in basic nursing and medical facilities. In 2010, the accessibility of CHs was highest in the suburban area (0.021), followed by the outskirt area (0.015), and lowest in the central area (0.008). By 2020, the suburban area still had the highest accessibility (0.016), the outskirt area remained stable (0.015), and the central area increased slightly (0.009). Overall, accessibility in the suburban area decreased over time, while the central area experienced a slight increase and the outskirt area remained essentially unchanged.

Finally, most towns/streets experienced changes in nursing care facility accessibility, with the highest-level towns/streets mainly gathered along the second ring expressway and extending gradually to the inner side of suburban areas and the outer side of outskirt areas. Medical facility accessibility decreased, with the highest-level towns/streets mainly concentrated close to the ring expressway and the second highest-level towns/streets mainly concentrated around the second ring expressway.

### 4.3 The supply-demand relationships of health care facilities

Before the exploration of supply-demand relationships, we analyzed the effect of two aging indices (aging density and degree) on the accessibility of health care facilities for older adults through multiple regression equations, with accessibility as the dependent variable and the two indices as the independent variables. As shown in Table 6, the indices of aging density and degree have a significant negative effect on accessibility in different years regardless of the type or level of health care facilities. The correlation results indicate the rationality of defining the supply-demand matching relationships on the basis of accessibility and the two aging indices.

**Table 6:** Coefficient of correlation between accessibility and indices of aging density and degree.

Variables		2000		2010		2020	
		Density	Degree	Density	Degree	Density	Degree
GHs		−0.135	−0.397^**^	−0.169^*^	−0.393^**^	−0.194^**^	−0.421^**^
MHs		−0.432^**^	−0.378^**^	−0.331^**^	−0.446^**^	−0.393^**^	−0.481^**^
CHs		null	null	−0.339^**^	−0.460^**^	−0.286^**^	−0.350^**^
HPs		null	null	null	null	−0.152^*^	−0.13
MFs	*p*	−0.135	−0.397^**^	−0.202^**^	−0.424^**^	−0.213^**^	−0.434^**^
	*t*	0.434^**^	−0.734^**^	0.256^*^	−0.615^**^	0.265^**^	−0.634^**^
NFs	*p*	−0.432^**^	−0.378^**^	−0.331^**^	−0.446^**^	−0.404^**^	−0.487^**^
	*t*	−0.347^**^	−0.109	0.002	−0.447^**^	−0.085	−0.423^**^
SFs	*p*	−0.193^*^	−0.423^**^	−0.227^**^	−0.430^**^	−0.292^**^	−0.476^**^
	*t*	0.336^**^	−0.683^**^	0.209^*^	−0.586^**^	0.152	−0.590^**^
CFs	*p*	null	null	−0.339^**^	−0.460^**^	−0.284^**^	−0.313^**^
	*t*	null	null	0.008	−0.466^**^	−0.112	−0.229^*^

MFs/NFs: medical facilities/nursing care facilities; SFs/CFs: state facilities/community-level facilities; p is the correlation coefficient, and t is the multiple linear regression factor coefficient; ^*^significance < 0.05, ^**^significance < 0.01.

Based on the division of supply-demand relationships, Table 7 shows the quantitative distribution of towns/streets categorized by their health care facility type and level in different geographical areas. In terms of different facility types, there is an undersupply of MFs in central areas due to the high aging population density and degree; moreover, the outskirt areas also have insufficient supply due to the high proportion of older adults in the total population. However, the opposite is true in suburban areas where older adults can fully access medical services. For NFs, the situation is similar to that of MFs, except for some oversupply in streets/towns on the outskirts. In terms of different facility levels, the quantitative distribution of SFs is the same as that of MFs; however, it is complicated for CFs. Specifically, the supply of CFs in central areas belongs to the bifactor-oriented undersupply type, but there is an oversupply in the suburban areas by contrast. The number of towns/streets with a high-level supply-demand balance and oversupply are both 14, which accounts for two-thirds of the total in the outskirts area.

**Table 7:** The number of towns/streets according to the supply-demand relationship of health care facilities in 2020.

Type	Health care facilities	Central area	suburban area	Outskirt area	Entire city
11	MFs	11	3	5	19
	NFs	0	4	12	16
	SFs	1	4	9	14
	CFs	10	3	14	17
21	MFs	1	37	10	48
	NFs	0	33	13	46
	SFs	0	38	12	50
	CFs	3	23	14	40
31	MFs	7	0	0	7
	NFs	8	0	0	8
	SFs	7	0	0	7
	CFs	6	0	1	7
32	MFs	5	1	20	26
	NFs	5	0	13	18
	SFs	5	0	16	21
	CFs	3	1	10	14
33	MFs	43	1	0	44
	NFs	53	1	0	54
	SFs	53	1	0	54
	CFs	47	1	0	48
41	MFs	14	7	11	32
	NFs	15	11	8	34
	SFs	15	6	9	30
	CFs	12	21	7	40

Overall, the city's supply is dominated by bifactor-oriented undersupply, supplemented by an excess supply of health care facilities; however, when analyzing the subdistricts, the supply of all types and levels of health care facilities for older adults is seriously inadequate. The suburbs, on the other hand, have an excessive supply, while the outskirts are dominated by degree-oriented supply deficiencies, supplemented by oversupply. The results show a high level of spatial variation in terms of the supply-demand relationships of health care facilities, which should be optimized in urban planning.

We further predicted population aging in the next 10, 20, and 30 years within cities. Table 8 presents the prediction results of older adults distributed in subdistricts in the next 30 years. The older population will continue to grow from 2020 to 2050, with an average of 1 million every ten years, and by 2050, older adults will reach 4,339,687 more than 3 times that in 2020. The distribution of older adults in the three subdistricts will reverse with a decrease from central to suburban and outskirt areas in 2020 and an increase from central to suburban and outskirt areas in 2050. The suburban and outskirt areas will experience faster population aging than the central area.

**Table 8:** Older population projections in three subdistricts from 2020 to 2050.

Sub district	2020	2030	2040	2050
Central area	668,999	859,088	1,137,797	1,304,391
suburban area	300,684	531,486	1,000,790	1,419,405
Outskirt area	355,474	708,262	1,181,236	1,615,890
Entire city	1,325,157	2,098,836	3,319,824	4,339,687

**Figure 7:**
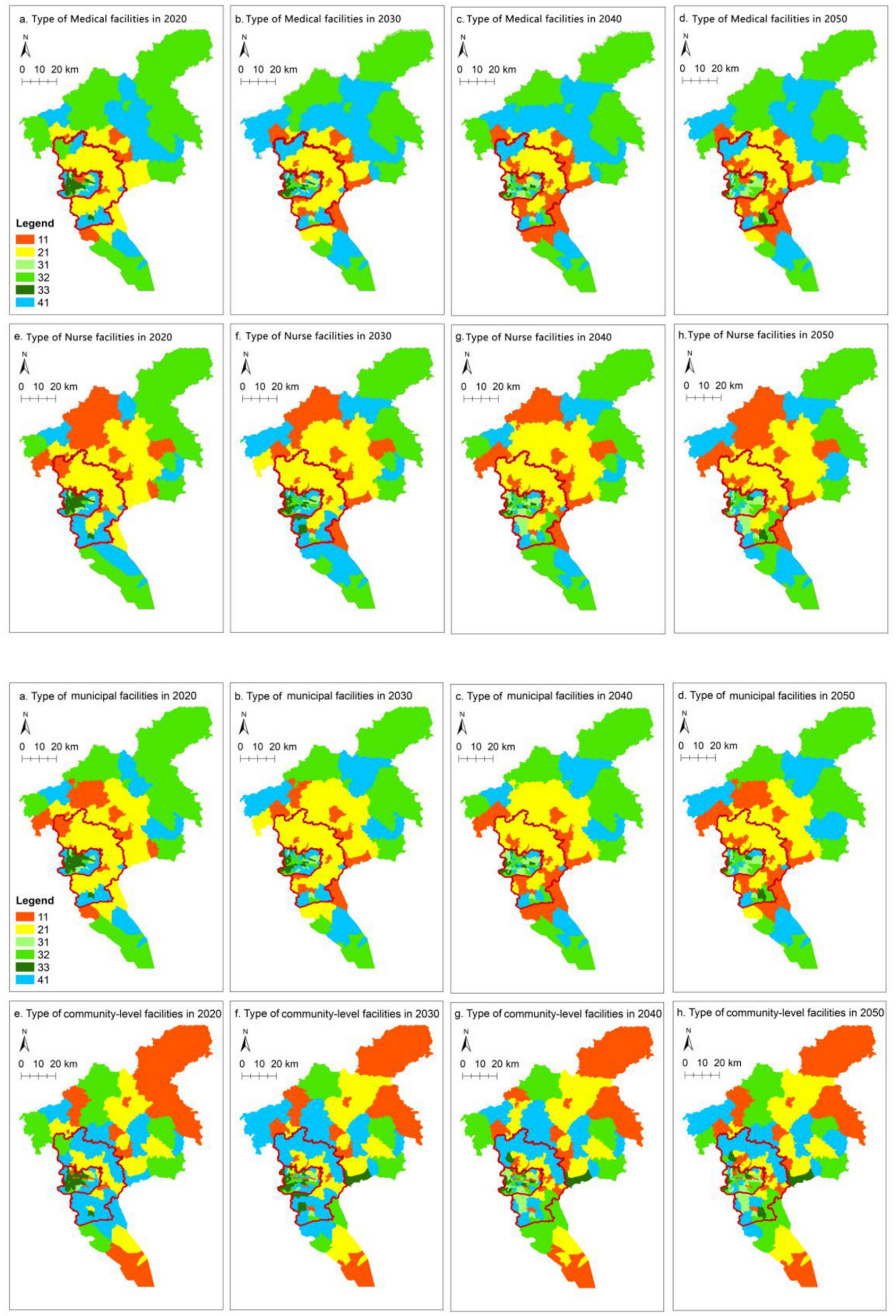
The spatial distribution of health care facilities divided by four categories.

To allocate health care resources for the next 30 years, a detailed analysis of the supply-demand relationship was conducted by categorizing supply and demand into six types, as introduced in the Methodology section. Table 9 shows the number of towns/streets according to facility type and level in different geographical areas, and Figure 7 displays the spatial distribution of health care facilities divided into four categories. In terms of different facility types, the supply of MFs will be deficient in the majority of towns/streets in the central area in the next 30 years, whereas the supply-demand relationship will mainly maintain oversupply in suburban areas and low demand on the outskirts. The distribution of NFs will be the same as that of MFs in central and suburban areas, while the demand on the outskirts will continuously increase along with the growing aging population and declining accessibility. When we investigated the distribution according to facility level, the situation will be similar to that based on their types in the central and suburban areas. However, the number of towns/streets will have little difference in various types (strong demand, oversupply, lagging supply, and low demand) in the outermost reaches of the city. For the last category of CFs, older adults in outskirt areas will have low demand in the coming three decades. In summary, the supply of health care facilities, regardless of the classification method, will change from sufficient in the next 10 years to a lagging state in 2040 and 2050. The central and suburban areas will show the opposite, with lagging supply in the former and oversupply in the latter. Moreover, the situation in the far suburbs will be more complex and volatile with diverse supply-demand relationships, which calls for more careful and detailed monitoring of the dynamic changes in different towns/streets.

**Table 9:** The number of towns/streets according to the supply-demand relationship of health care facilities in 2030/2040/2050.

Year	Type	Health care facilities	Central area	Suburban area	Outskirt area	Entire city
2030	11	MFs	7	8	7	22
		NFs	0	6	12	18
		SFs	1	8	8	17
		CFs	2	6	12	26
	21	MFs	5	32	8	45
		NFs	0	31	13	44
		SFs	0	34	13	47
		CFs	5	20	16	41
	31	MFs	8	1	0	9
		NFs	8	1	0	9
		SFs	8	1	0	9
		CFs	5	1	1	7
	32	MFs	21	1	13	35
		NFs	26	1	8	35
		SFs	26	1	12	39
		CFs	22	1	6	29
	33	MFs	25	0	0	25
		NFs	27	2	0	29
		SFs	26	0	0	26
		CFs	26	2	1	29
	41	MFs	15	7	18	40
		NFs	20	8	13	41
		SFs	20	5	13	38
		CFs	15	19	10	44
2040	11	MFs	11	16	10	37
		NFs	0	12	13	25
		SFs	1	16	11	28
		CFs	10	11	13	34
	21	MFs	1	24	5	30
		NFs	0	25	12	37
		SFs	0	26	10	36
		CFs	3	15	15	33
	31	MFs	21	1	0	22
		NFs	22	4	0	26
		SFs	22	1	0	23
		CFs	18	4	1	23
	32	MFs	23	2	15	40
		NFs	31	3	12	46
		SFs	30	2	14	46
		CFs	25	3	10	38
	33	MFs	13	0	0	13
		NFs	15	0	0	15
		SFs	14	0	0	14
		CFs	15	1	1	17
	41	MFs	12	6	16	34
		NFs	13	5	9	27
		SFs	13	4	11	28
		CFs	10	15	6	31
2050	11	MFs	11	18	8	37
		NFs	0	14	14	28
		SFs	1	18	11	30
		CFs	11	12	12	35
	21	MFs	1	22	7	30
		NFs	0	23	11	34
		SFs	0	24	10	34
		CFs	2	14	16	32
	31	MFs	22	1	0	23
		NFs	23	4	0	27
		SFs	23	1	0	24
		CFs	19	4	1	24
	32	MFs	25	2	14	41
		NFs	32	3	8	43
		SFs	32	2	11	45
		CFs	25	4	8	37
	33	MFs	7	1	0	8
		NFs	10	1	0	11
		SFs	9	1	0	10
		CFs	10	2	1	13
	41	MFs	15	5	17	37
		NFs	16	4	13	33
		SFs	16	3	14	33
		CFs	14	13	8	35

## 5 Discussion

Similar spatial disparities in elderly healthcare accessibility have been identified in previous studies. Niu et al. (21)highlighted significant spatiotemporal variations in emergency medical accessibility in Zhengzhou driven by temporal and traffic conditions. In contrast, this study focuses on integrated medical–nursing facilities in Guangzhou and reveals that spatial inequity is more closely associated with hierarchical heterogeneity in service provision within a complex multi-centric urban system. These findings suggest that, beyond temporal dynamics, structural differences in healthcare systems are also a key driver of accessibility inequality. This study investigated and analyzed the spatial distribution of the aging population and accessibility of health care facilities for older adults in Guangzhou were conducted. Several points need to be considered when interpreting the results of this study. First, from the perspective of geographical location, due to the high proportion of older inhabitants, it is difficult to meet the requirements of an aging society in the central area of the city, where accessibility is always lower than that in the other two subdistricts. This result is in contrast to those of other studies on Guangzhou's residential care facilities and basic medical services (27). The possible reason for this contradiction is the difference in the delineation of the geographic area. In other studies, there is no subdivision of the whole city, which was subdivided to compare the differences in accessibility of three subareas. Other studies have analyzed accessibility from an overall spatial scale, resulting in a high central low peripheral health care distribution pattern. Moreover, accessibility in the central part of the city showed a downward trend in medical facilities, whereas the nursing facilities that are mainly based on utilizing existing buildings experienced an upward trend.

Second, the suburban area has an aging population that is increasing fast but from a small base. Therefore, people's demands can be effectively met, and health care accessibility is high, which results in the best situation of the three subdivided geographic areas. Moreover, the supply-demand relationships of the past and future indicate an oversupply. In particular, this area is surrounded by two transportation circuits (ring expressway and second ring expressway), which provide convenient access to health care facilities. Therefore, the urgency of allocating additional facilities is lower than in other areas. In the future, as the suburban older adults continues to grow, the allocation strategy should emphasize incremental, community-level interventions rather than large-scale expansion. It is aligns with changing demographic patterns and demand dynamics (36). Moreover, periodic reassessment of the supply–demand balance and transportation development remains essential for adaptive planning.

Third, the situation on the outskirts is the most complicated, with the largest number of older adults due to the vast geographical coverage, but the proportion is not high and the increase in the aging population is slow. However, due to the different functional positioning and land use planning of different subdivided towns/streets in this subdistrict, the accessibility and supply-demand relationships vary greatly. Specifically, many older adults are willing to choose to live there due to the large-scale communities with pleasant living environments or complete living facilities. These concentrated populations eventually bring about a shortage in the supply of health care facilities. Some streets and towns with a smaller aging population are dominated by low demand. Along with the growing aging population, an increasing number of older adults who prefer favorable air quality and pleasant living environments will choose to live in remote areas to escape from the noise of urban life, and thus, future supply-demand relationships on the outskirts will gradually shift from low demand to high demand. In these circumstances, rather than increasing the supply of health care facilities on a large scale, attention should be given to the planning and construction of key areas in the future.

Finally, from the perspective of different classifications, accessibility is higher for medical facilities than for nursing facilities, but the former is experiencing a downward trend and the latter an upward trend. Similarly, the accessibility of municipal-level facilities, which is experiencing a downward trend, is higher than that of community-level facilities. This result is consistent with the research of J. Li et al. (27) on different levels of health care facilities in Guangzhou. However, they only used data from a single year, 2020, and did not analyze the trend. This is because, with the implementation of the integrated health care policy, community-level facilities and nursing facilities, which cover a small area and can be renovated based on existing buildings, dominate the new supply of health care facilities and have substantially increased in recent years. On the other hand, as urban construction land becomes increasingly scarce, medical facilities, especially municipal facilities that occupy a large area, are more difficult to construct within a short period of time. In the future, the allocation of health care facilities should focus on a balance between different types and actively develop small-scale community-based hospitals by taking full advantage of existing buildings.

By identifying poorly suited geographical areas and the types of facilities that are lacking, we considered that the space and land resources in different geographical areas are varied and proposed a dispersal strategy that is feasible to implement. Specifically, there are four recommendations for urban planners and policy-makers. First, the municipal government urgently needs to balance different districts and categories of health care facilities by embracing more private and market forces and increasing public health care investment to reduce the gaps identified in this research. Second, it is recommended to improve the transportation conditions in areas with low accessibility by connecting them with main traffic arteries. For example, on the edge of the central city area and outskirts, it would be a possible solution to open new bus routes linking communities with health care facilities near entrances and exits of the ring expressway and second ring expressway. Third, more private care institutions operated could be actively encouraged and built for the development of integrated health care by providing policy incentives, financial aid, and standard training for caregivers. Especially for central areas with space shortages, it is more important to fully exploit the potential of existing health care facilities and improve their service capability by management restructuring. For example, those with a decent foundation could have a unified administration and management through standardized criteria to achieve good coordination and complementation between public and private facilities.

Finally, we recommend that policy-makers give priority to expanding the scale of existing facilities to meet the needs of older adults. This solution would be more suitable for the situation in outskirt areas that have relatively sufficient construction land, such as Nansha District and Zengcheng District. Considering the complex condition of the outskirts as previously analyzed, it is important to have a community-level analysis and expand or build new health care facilities in the areas where accessibility is low and supply is lagging. Recent studies similarly emphasize that suburban and peri-urban areas consistently suffer from lower healthcare accessibility due to structural supply–demand mismatches and uneven spatial distribution of services (37, 38). Especially for those areas with large-scale residential communities or near the second ring expressway, expansion or construction should be the top priority for urban planners and public officials. Large residential communities concentrate older adults and other high-demand groups, making them priority spatial targets for facility provision, while locations near major transport nodes enable new facilities to function as networked service hubs serving multiple surrounding neighborhoods and emerging peri-urban clusters, thereby improving both efficiency and spatial equity (39).

## 6 Conclusion

With the increase in human life quality and expectancy and the decline in fertility rates, the aging population is becoming a serious issue for many countries worldwide. As one of the most populous countries, China is also facing the challenges of an aging population, with a growing number of older citizens requiring health care and long-term care services. The government has recognized the importance of supplying health care and support for older adults in their homes and local communities. Therefore, the integrated health care policy, which combines medical and nursing health care services by providing home and community-based facilities for older adults, was introduced in recent years. However, previous studies have not sufficiently evaluated the practice of this policy, especially its spatial allocation of community-based health care facilities. This research fills this gap from the perspective of urban geography and urban planning.

This study contributes to the theoretical understanding of healthcare accessibility by explicitly integrating multi-level and multi-type medical–nursing facilities into spatial equity analysis. While previous studies mainly focus on single-type facilities or general population accessibility, our approach highlights the hierarchical heterogeneity and interactions among different types of facilities within complex urban systems. Specifically, first, rather than overlooking the hierarchical and heterogeneous nature of different health care facilities, we analyzed the spatial accessibility of health care facilities in a metropolis in the past 20 years and the next three decades by accounting for their various levels and categories. Second, we revealed a result contrary to that of previous studies in Guangzhou, where the central city has suffered low accessibility and undersupply due to the large density of the older population and the lack of available land for newly built facilities (10). This difference may be attributed to the exceptionally high density of older adults in central districts, the limited opportunities for facility expansion, and the fact that demand growth outpaces service capacity (40). This should inform policy-makers as to the further allocation of health care resources, especially medical facilities in central areas, by encouraging private care institutions and fully exploiting the potential of existing buildings or facilities.

The research findings in this article suggest a dispersal strategy to improve the spatial accessibility and supply-demand relationships of health care facilities in Guangzhou. Generally, both medical and nursing facilities in central areas should be the top priority by fully exploiting existing resources, while new facilities should be appropriately constructed in periphery areas, such as the outskirts of Nansha and Zengcheng districts, especially those large-scale residential communities.

Despite the strengths of our research, there are also some limitations. First, the service distance for each type of health care facility for calculating accessibility was a linear distance rather than the shortest passable distance, which may reduce the reliability and explanatory power of the results. In addition, the use of predefined travel-time thresholds and average travel-speed assumptions, together with the absence of sensitivity analysis and older adults' mobility, may introduce some uncertainty into the accessibility estimates. Other researchers could attempt other modified calculation methods in the future. Second, the analysis unit of the aging population and geographical areas in this study was the town/district level, which was not sufficient for the analysis of outskirt areas where the situation is complicated. However, considering the availability of community-level population data, this level was the smallest geographical unit to evaluate the accessibility of the whole city. Further research could be conducted to obtain a more detailed analysis of the supply-demand relationships in outskirt areas if there is access to the data. In addition, due to data availability, healthcare service capacity was represented by the number of beds only; future studies could include additional supply-side indicators to provide a more comprehensive assessment.

## Data Availability

The data analyzed in this study is subject to the following licenses/restrictions: The dataset is available from the corresponding author upon reasonable request. Requests to access these datasets should be directed to wuyifei@szpu.edu.cn.

## Footnotes

1 Home-based care providers offer a range of services, including nursing care, personal care assistance, medication management, rehabilitation, and palliative care, delivered in individuals' homes. These services aim to support individuals with activities of daily living and help them maintain their independence.

2 China has established daycare centers, also known as community care centers or community-based care centers, where older adults can receive various services during the day. These centers offer medical care, rehabilitation, social activities, and support services, allowing older adults to interact with their peers and receive care in a community setting.

3 China has established a vast network of community health centers to deliver primary health care services at the grassroots level. These community health centers play a vital role in providing home-based care, health education, disease prevention, and management services to the local population, including older adults.
